# Systematization and Comparison of the Binary Successive Approximation Variants

**DOI:** 10.3390/s21248267

**Published:** 2021-12-10

**Authors:** Konrad Jurasz, Dariusz Kościelnik, Jakub Szyduczyński, Marek Miśkowicz

**Affiliations:** 1 Department of Electronics, AGH University of Science and Technology, 30-059 Kraków, Poland; koscieln@agh.edu.pl (D.K.); szyduczy@agh.edu.pl (J.S.); 2 Department of Measurement and Electronics, AGH University of Science and Technology, 30-059 Kraków, Poland; miskow@agh.edu.pl

**Keywords:** successive-approximation, analog-to-digital conversion, time-to-digital conversion

## Abstract

This paper presents a systematization and a comparison of the binary successive approximation (SA) variants. Three different variants are distinguished and all of them are applied in the analog-to-digital conversion. Regardless of an analog-to-digital converter circuit solution, the adoption of the specific SA variant imposes a particular character of the conversion process and related parameters. One of them is the ability to direct conversion of non-removeable physical quantities such as time intervals. Referencing to this aspect a general systematization of the variants and a name for each of them is proposed. In addition, the article raises the issues related to the complexity of implementation and energy consumption for each of the discussed binary SA variants.

## 1. Introduction

The successive approximation (SA) method is known at least from the 16th century [1]. One of its most common applications was conversion weight of an object to numbers, which in fact is simple analog-to-digital conversion already.

The first implementation of the binary SA scheme in electronic analog-to-digital conversion dates back to the 1950s. Developed for decades it has become fundamental and one of the most successful analog-to-digital conversion techniques. Nowadays, it is usually chosen as compromise between fast but expensive flash and precise but slow integrating analog-to-digital conversion method [1,2,3].

From the very first SA analog-to-digital converter (ADC) all of the solutions based on one of the three binary SA variants. However, in most cases the clear statement referencing to applied SA variant is omitted. Moreover, in literature the SA method itself is often incorrectly identified with only one SA variant. This is possibly caused by a lack of systematization. Therefore, based on the most distinctive parameters of the binary SA variants, a name for each of them is proposed.

Each of the three SA variants has specific properties and one of them is the ability to direct conversion of some non-removable physical quantities such as time intervals. There are cases, where it plays a crucial role and its lack can be an inconvenient obstacle or even disqualifying limitation [4]. If only for this reason, the awareness of differences between the SA variants is important, because it allows to choose the most appropriate one for a specific application.

The following sections are focused especially on the systematization and naming of the SA variants based on the above-mentioned ability to direct conversion of some physical quantities. In addition, the issues of energy consumption and complexity of implementation are discussed.

## 2. The Binary Successive Approximation Variants

**Figure 1:**
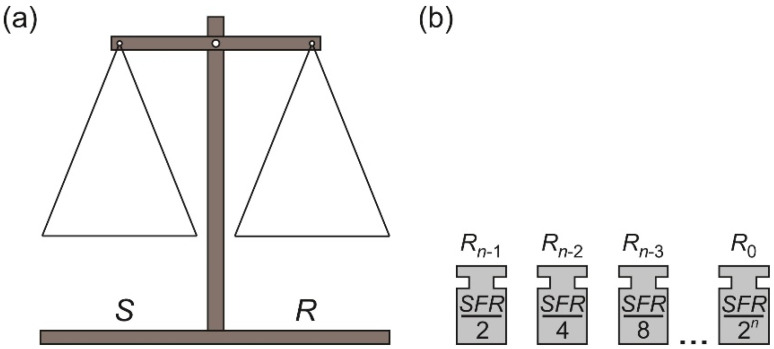
The model of the binary SA conversion system: (**a**) the pan balance model; (**b**) the binary-scaled reference elements.

In general, the binary SA method approximates the measured input value *X_IN_* with an appropriate subset of predefined, binary-scaled reference units. The unique character of the SA is derived from a very well-known weighing method, which uses a pan balance and a set of reference elements. This is one of the reasons why it is convenient to illustrate the binary SA method as a weighing process. Such an approach is also used in the following analysis in order to explain the differences between the presented algorithms. Both the pan balance and the reference elements are shown in Figure 1.

The used model of the balance consists of the source pan *S* and the reference pan *R* (Figure 1a). The binary-scaled reference elements *R_n_*_−1_, …, *R*_0_ (Figure 1b) are placed on the pans in order to accomplish appropriate binary SA algorithm. The measured input value *X_IN_* is always placed on the pan *S*, but the placement of the reference elements *R_n_*_−1_, …, *R*_0_ depends on the applied SA variant.

The values of the reference elements *R_n_*_−1_, …, *R*_0_ are defined as *R_k_ =* 2*^k^R*_0_, for *k* = 0, 1, …, *n* − 1. Obviously, there is an accurate relation between the reference elements *R_n_*_−1_, …, *R*_0_ and the parameters of the pan weighing. First of all, with *n* reference elements *R_n_*_−1_, …, *R*_0_ it is possible to represent the measured input value *X_IN_* as one of the 2*^n^* different subsets of the reference elements *R_n_*_−1_, …, *R*_0_. Moreover, the total weight of the subsets can vary in range 〈0, *R*_0_, …, (2*^n^* − 1)*R*_0_〉. Secondly, because of the finite number of the reference elements *R_n_*_−1_, …, *R*_0_, the resolution of the conversion process is also finite.

Considering the above, the input weight *X_IN_* is always measured with determined resolution *R*_0_. In addition, it has to be less than 2*^n^R*_0_ in order to be properly measured. This limiting value can be termed as *signal full range* (*SFR*):

(1) $$SFR=2^{n}R_{0}.$$

### 2.1. Oscillating Successive Approximation

The first variant of the binary SA method requires one set of the reference elements *R_n_*_−1_, …, *R*_0_. Their placement is limited to the pan *R* only.

**Figure 2:**
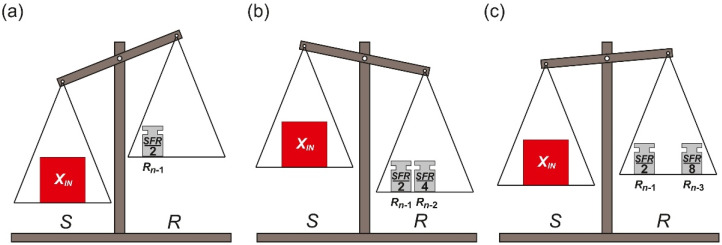
Illustration of oscillating successive approximation steps: (**a**) the first step; (**b**) the overestimation; (**c**) compensation of the overestimation.

The conversion process starts from placing the measured input element *X_IN_* on the pan *S*. Simultaneously, the biggest reference element *R_n_*_−1_ is placed on the pan *R* (Figure 2a). At each next step, until the smallest reference element *R*_0_ is used, two operations are performed. Firstly, the current state of the pan balance is analyzed. If the total weight of the reference elements currently placed on the pan *R* is not greater than the weight of the measured input value *X_IN_* (Figure 2a), the most recently placed reference element *R_k_* is left on the pan *R*. Otherwise, the most recently placed reference element *R_k_* is removed from the pan *R* (Figure 2b,c), which means a change of the previously made decision. Secondly, in both cases, twice smaller reference element *R_k_*_−1_ (*k* ≥ 1) is placed on the pan *R*.

The last step of the conversion process is made after the placement of the smallest reference element *R*_0_ on the pan *R*. It is limited only to decision if the reference element *R*_0_ should be removed from the pan *R* or not.

When the conversion process is completed the measured input value *X_IN_* is approximated by an appropriate subset of the reference elements which are left on the pan *R*. Thus, the final result of the conversion process can be expressed as:

(2) $$X_{IN}\approx \overset{n-1}{\underset{k=0}{\sum }}\left(R_{k}\cdot P_{k}\right),$$

where *P_k_* indicates if the *k*-th reference element *R_k_* has been left on the pan *R* (*P_k_* = 1) or not (*P_k_* = 0).

**Figure 3:**
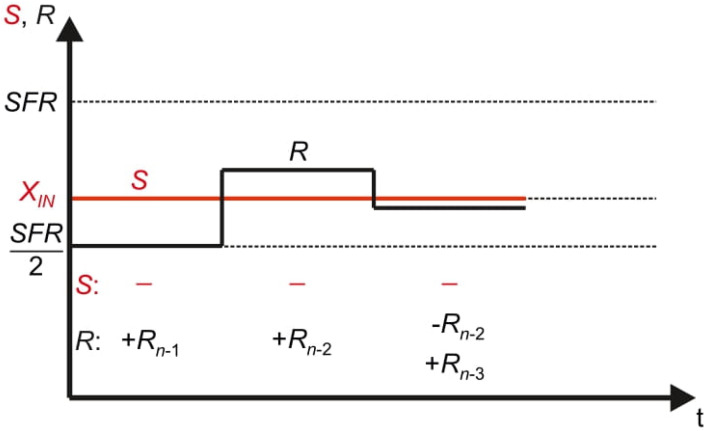
Oscillating successive approximation process in the time domain.

Figure 3 presents the conversion process in the time domain. Performed operations of adding and optional removing the reference elements *R_n_*_−1_, …, *R*_0_ from the pan *R* cause oscillations around the measured input value *X_IN_*. Therefore, the name *oscillating successive approximation* (OSA) is proposed for this algorithm [5].

**Figure 4:**
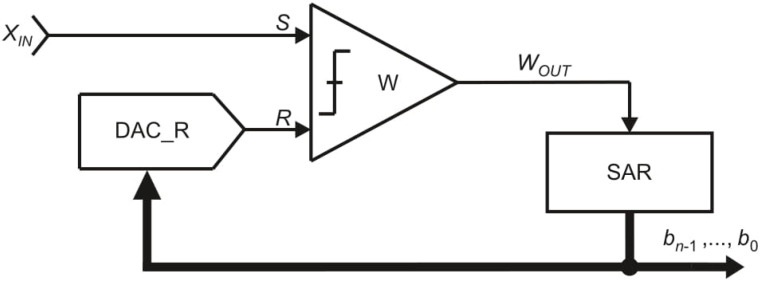
Simplified model of the oscillating successive approximation converter.

The model of the OSA converter is shown in Figure 4. It consists of one comparator *W*, one digital-to-analog converter DAC_R and a control logic block SAR (Successive Approximation Register). The inputs of the comparator W, signed as *S* and *R*, refer accordingly to the pans *S* and *R*. The digital-to-analog converter DAC_R in reference path generates the reference signal *R*. The comparator *W* compares the signals *S* and *R* and indicates to the control logic SAR the relation between them. Based on this information the control logic decides if the reference signal *R* should be reduced or not.

The equivalent of the measured input value *X_IN_* is represented by the *n*-bit digital output word *b_n_*_−1_, …, *b*_0_. At the *i*-th step of the conversion process, for *i* = 2, 3, …, *n* + 1 (except the first step), one bit *b_i_* is evaluated, starting from the most significant bit *b_n_*_−1_. If the reference signal *R* is not greater than the source signal *S*, the bit *b_n_*_−*i* + 1_ is set to logic “1”. Otherwise, the bit *b_n_*_−*i* + 1_ is set to logic “0”.

One of the most distinctive features of the OSA variant is necessity of removing specific reference element *R_k_* (Figure 2c) in case of overestimation (Figure 2b). This subtraction means a change of the previously made decision. Obviously, such operation is not always possible during the conversion process. Considering a specific case, when the measured input value *X_IN_* is non-removable, non-decremental physical quantity, the reference elements *R_n_*_−1_, …, *R*_0_ are also non-decremental. An example of such measured physical quantity *X_IN_* is time interval for which the reference elements *R_n_*_−1_, …, *R*_0_ are also non-decremental time units. In such case the time reference elements *R_n_*_−1_, …, *R*_0_ cannot be directly removed during conversion, because it is impossible to turn back time. Nevertheless, it should be noted that indirect conversion of non-removable values is still possible using the OSA algorithm [6]. It requires an additional preconversion process to replace non-removable value *X_IN_* by removable physical quantity (e.g., charge or voltage).

### 2.2. Full-Scale Monotonic Successive Approximation

**Figure 5:**
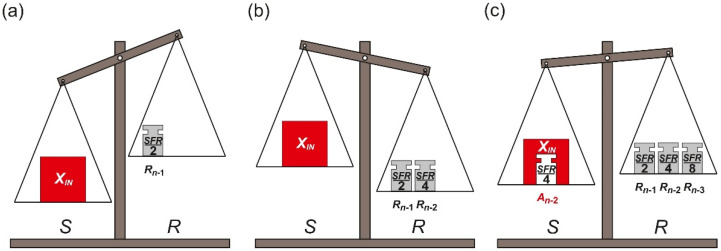
Illustration of full-scale monotonic successive approximation steps: (**a**) the first step; (**b**) the overestimation; (**c**) compensation of the overestimation.

The necessity of removal operation, which is associated with the OSA algorithm, does not occur in the second SA variant. The problem is solved by an additional set of binary-scaled reference elements *A_n_*_−1_, …, *A*_0_ defined as *A_k_* = 2*^k^A*_0_ (Figure 5—white reference weights). Each additional reference element *A_k_* from the set *A_n_*_−1_, …, *A*_0_ is equal to the appropriate reference element *R_k_* (*A_k_* = *R_k_*) from the set *R_n_*_−1_, …, *R*_0_ (Figure 5—gray reference weights). Similarly to the OSA, the reference elements *R_n_*_−1_, …, *R*_0_ can be placed only on the pan *R*, but the additional reference elements *A_n_*_−1_, …, *A*_0_ can be placed only on the pan *S*.

The conversion process starts when the measured input element *X_IN_* is placed on the source pan *S*. Simultaneously, the biggest reference element *R_n_*_−1_ is placed on the pan *R* (Figure 5a). At each next step, until the smallest reference element *R*_0_ is used, two operations are performed. Firstly, the current state of the pan balance is analyzed. If the total weight of the reference elements currently placed on the pan *R* is not greater than the total weight of the elements currently placed on the pan *S* (Figure 5a), no correction is needed at this step. Otherwise, the most recently placed reference element *R_k_* is compensated by the appropriate additional reference element *A_k_* placed on the pan *S* (Figure 5b,c). Secondly, in both cases, subsequent reference element *R_k_*_−1_ (*k* ≥ 1) is placed on the pan *R*.

When the smallest reference element *R_0_* is used, the conversion is at its final step. The last operation required to accomplish the conversion process is a decision whether to add the additional reference element *A*_0_ on the pan *S* or not.

It clearly follows from the above that all of the reference elements *R_n_*_−1_, …, *R*_0_ are successively placed on the pan *R* and none of them is removed. On the other hand, the additional reference elements *A_n_*_−1_, …, *A*_0_ complement the measured input value *X_IN_* with the assumption that the total weight on the source pan *S* has to be contained in range 〈*SFR − R*_0_, *SFR*). Thus, the measured input value *X_IN_* can be evaluated as the difference between the subsets of the reference elements and the additional reference elements placed on the pans:

(3) $$X_{IN}\approx \overset{n-1}{\underset{k=0}{\sum }}R_{k}-\overset{n-1}{\underset{k=0}{\sum }}\left(A_{k}\cdot P_{k}\right),$$

where *P_k_* indicates if the *k*-th additional reference element *A_k_* has been placed on the pan *S* (*P_k_* = 1) or not (*P_k_* = 0). Moreover, at the end of the conversion process the total weight on the pan *R* always equals (*SFR* − *R*_0_), so the Equation (3) can be rewritten as:

(4) $$X_{IN}\approx SFR-R_{0}-\overset{n-1}{\underset{k=0}{\sum }}\left(A_{k}\cdot P_{k}\right)$$

It should be noted that the subtraction in the Equations (3) and (4) is not necessary in order to obtain the correct result. The equivalent of the measured input value *X_IN_* can also be expressed as the additional reference elements from the set *A_n_*_−1_, …, *A*_0_, which remained unused (do not complement the input value *X_IN_* on the pan *S*) at the end of the conversion process:

(5) $$X_{IN}\approx \overset{n-1}{\underset{k=0}{\sum }}\left(A_{k}\cdot {\bar{P}}_{k}\right).$$

**Figure 6:**
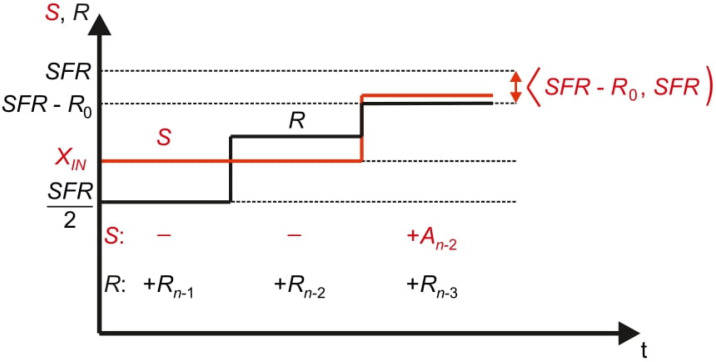
Full-scale monotonic successive approximation process in the time domain.

The algorithm ensures that the total weight of each of the pans can only increase, so the removal operation (change of the previously made decision) is unnecessary. The total weight of the elements placed on the pan *R* will monotonically approach to (*SFR* − *R*_0_) while the total weight of the elements placed on the pan *S* will be complemented in order to be contained between (*SFR* − *R*_0_) and *SFR* (Figure 6). Relating to this specific, monotonic character of the functions the name *full-scale monotonic successive approximation* (FSMSA) is proposed for this algorithm.

**Figure 7:**
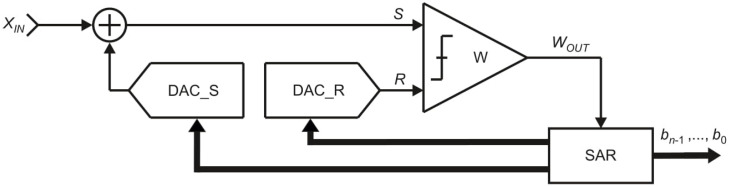
Simplified model of full-scale monotonic successive approximation converter.

The simplified model of the FSMSA algorithm consists of one comparator W, two digital-to-analog converters—DAC_R and DAC_S—and a control logic block SAR (Figure 7). Similar to the OSA, the digital-to-analog converter DAC_R represents the behavior of the pan *R*. The additional digital-to-analog converter DAC_S generates the component of the source signal *S*, which complement the measured input value *X_IN_*. The comparator W indicates to the control logic SAR current relation between the source signal *S* and the reference signal *R*. Based on this information, the control logic SAR decides whether the value of the source signal *S* should be increased or not.

In the FSMSA variant of the SA method, similarly to the OSA, the digital equivalent of the measured input value *X_IN_* is represented by the *n*-bit digital output word *b_n_*_−1_, …, *b*_0_. During the conversion process, based on the output value *W_OUT_* of the comparator W, the subsequent bits *b_n_*_−1_, …, *b*_0_ are evaluated. At the *i*-th step of the conversion process (*i* = 2, 3, …, *n* + 1), if the reference signal *R* is not greater than the source signal *S*, the bit *b_n_*_−*i* + 1_ is set to logic “1”. Otherwise, the bit *b_n_*_−*i* + 1_ is set to logic “0”.

As it has been shown, the FSMSA variant does not require removal operation. It means that once made decision does not have to be changed in order to acquire the right equivalent of the measured input value *X_IN_*. This feature was achieved, among others, by using the additional set of the reference elements *A_n_*_−1_, …, *A*_0_. When the measured input value *X_IN_* is overestimated (*S* < *R*), the appropriate element from the additional reference set *A_n_*_−1_, …, *A*_0_ is used for compensation.

Lack of the removal operation allows for direct conversion of non-removable values such as time intervals. In the FSMSA variant the measured time interval *X_IN_* need only to be increased by the additional reference elements *A_n_*_−1_, …, *A*_0_ defined in the time domain. It allows to avoid the necessity of removal operation, which is impossible in case of time measurement. Of course, the usage of the additional reference elements *A_n_*_−1_, …, *A*_0_ by definition causes an increase in the hardware resources and energy consumption. Nevertheless, the advantage over OSA in the direct conversion ability causes that there are solutions based on the FSMSA variant [7].

### 2.3. Monotonic Successive Approximation

The third successive approximation algorithm requires, as in case of the OSA, only one set of the reference elements *R_n_*_−1_, …, *R*_0_ to approximate the measured input value *X_IN_*. However, similarly to the FSMSA, the reference elements *R_n_*_−1_, …, *R*_0_ can be placed on both pans: *S* and *R*. The practical consequence of having just one set of the reference elements *R_n_*_−1_, …, *R*_0_ in combination with the fact that they can be distributed on both pans (*S* and *R*) is that a given weight *R_k_* can be used for both estimation (pan *R*) and compensation of overestimation (pan *S*).

**Figure 8:**
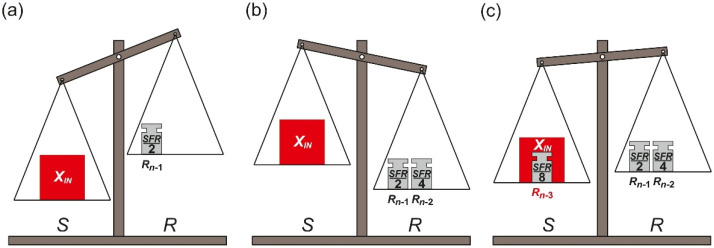
Illustration of monotonic successive approximation steps: (**a**) the first step; (**b**) the overestimation; (**c**) compensation of the overestimation.

The conversion process starts when the measured input element *X_IN_* is placed on the source pan *S*. Simultaneously, the biggest reference element *R_n_*_−1_ is placed on the reference pan *R* (Figure 8a). At each next step, the subsequent reference element *R_k_* (*k* < *n* − 1) is placed on the pan (*S* or *R*), on which currently accumulated elements weigh less (Figure 8b,c).

The last step of the conversion process is performed once the last reference element *R_0_* is placed on one of the pans. It is limited only to determining the final deflection of the pan balance. This information is used to determine the final result of the conversion process.

**Figure 9:**
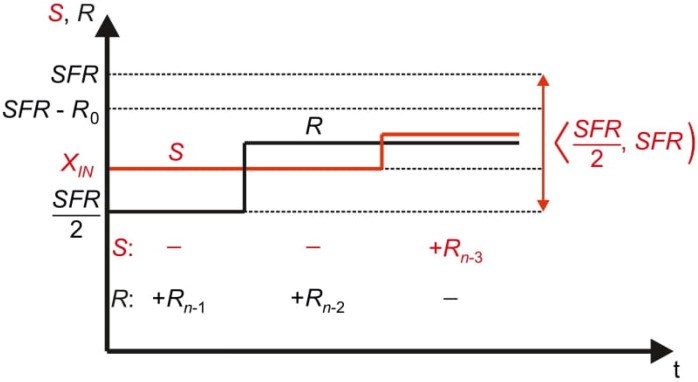
Monotonic successive approximation process in time domain.

Despite the fact that only one set of the reference elements is used in the conversion process, the removal operation (change of the previously made decision) is unnecessary. The reference elements *R_n_*_−1_, …, *R*_0_ are only added, so the total weight of each pan can only increase. Moreover, during the conversion process, the total weight of each pan approaches to each other monotonically (Figure 9). That is why the name *monotonic successive approximation* (MSA) is proposed for this algorithm.

The conversion character of the FSMSA and the MSA variants in some cases may be similar (Figure 6 and Figure 9). Nevertheless, the essential difference of using only one set of the reference elements *R_n_*_−1_, …, *R*_0_ in the MSA causes that the total weight accumulated on each of the pans at the end of the conversion process is always contained between *SFR*/2 and *SFR*. The range is relatively wide in comparison to the FSMSA. In that algorithm the total weight accumulated on the pan *S* at the end of the conversion process varies between (*SFR* − *R*_0_) and *SFR*, while the total weight on the pan *R* is equal to (*SFR* − *R*_0_).

At the end of the conversion process the MSA algorithm provides balance between the pans (*S* and *R*) with assumed resolution *R*_0_. Including the information about the final deflection of the pan balance, the equivalent of the measured input value *X_IN_* can be evaluated as:

(6) $$X_{IN}\approx \left(\overset{n-1}{\underset{k=0}{\sum }}\left(R_{k}\cdot P_{k}\right)\right)-\left(\overset{n-1}{\underset{k=0}{\sum }}\left(R_{k}\cdot {\bar{P}}_{k}\right)\right)-R_{0}\cdot Q,$$

where *P_k_* indicates if the *k*-th reference element has been placed on the pan *R* (*P_k_* = 1) or not (*P_k_* = 0). The final deflection of the pan balance is included in the Equation (6) with *Q*. If the total weight of the reference elements placed on the pan *R* is not greater than the total weight of the elements placed on the pan *S*, *Q* equals 0. Otherwise, *Q* equals 1. It should be noted that as in previous binary SA algorithms the digital representation of the measured input value *X_IN_* can be successfully obtained without this subtraction [8], which may be suggested by the Equation (6).

**Figure 10:**
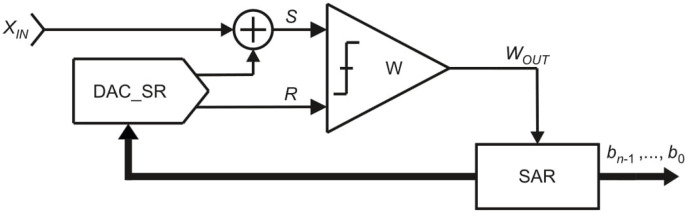
Simplified model of the monotonic successive approximation.

The simplified model of the MSA is presented in Figure 10. It consists of: one comparator W, one digital-to-analog converter DAC_SR with two outputs and a control logic block SAR. Similarly to the previous SA algorithms, the inputs *S* and *R* of the comparator W refer accordingly to the pans *S* and *R*. The digital-to-analog converter DAC_SR generates the values for both the source signal *S* and the reference signal *R*. Based on the output value of the comparator W, the control logic SAR decides which signal: *S* or *R* should be increased in the next step.

Similarly to the models of the previously presented SA algorithms, the bits in the digital output word *b_n_*_−1_, …, *b*_0_ are evaluated successively, starting from the most significant bit *b_n_*_−1_. At the *i*-th step of the conversion process (*i* = 2, 3, …, *n* + 1), if the reference signal *R* is not greater than the source signal *S* the bit *b_n_*_−*i* + 1_ is set to logic “1”. Otherwise, the bit *b_n_*_−*i* + 1_ is set to logic “0”.

The MSA algorithm neither requires removal operation (change of the previously made decision) nor the additional set of the reference elements (compensation of the previously made decision). This specific feature is achieved by relatively more complex structure of the circuit (Figure 10). Unnecessity of the removal operation allows for direct conversion of non-removable physical quantities. Therefore, this binary SA algorithm is often used in Time-to-Digital Converters [5,9].

## 3. Comparison of the Binary Successive Approximation Variants

In the previous sections the description of the binary SA algorithms focused mainly on the ability to direct conversion of non-removable physical quantities. Without a doubt it is a very important aspect of the analog-to-digital conversion, especially from the perspective of time measurement. However, presented models permit the distinction of more differences between presented binary SA algorithms, which also strongly affect the conversion process.

In reference to the previous sections, one of the most evident parameters concerns the number of steps required to determine the equivalent of the measured input value *X_IN_*. Obviously, it is directly related to the number of the reference elements *R_n_*_−1_, …, *R*_0_. In all presented binary SA algorithms the number of steps equals (*n* + 1) for *n* used reference elements *R_n_*_−1_, …, *R*_0_. Nevertheless, each of the SA algorithms performs different operations within a single step and it must be taken into consideration in order to compare the algorithms reliably.

First of all, the binary SA algorithms use different types of mathematical operations. The OSA uses two types: necessary addition and optional subtraction. For non-removable physical quantities, the optional subtraction, used in case of overestimation (*S* < *R*), limits this SA algorithm to indirect conversion only. Of course, possibility of overestimation of the measured input value *X_IN_* is an inherent factor of the binary SA and the subtraction is the simplest way to overcome the problem. However, in order to convert directly non-removable values such as time intervals, the subtraction operation cannot occur directly in the SA algorithm.

Eliminating of subtraction results in addition operation (“(−1) (−1) = (+1)”), so in the FSMSA algorithm, instead of optional subtraction, additional operation of addition is used. In result, the FSMSA uses only addition operations during the conversion process, which allows for direct conversion of non-removable values.

Two addition operations can be replaced by one addition operation (“(+1) (+1) = (+1)”). This is applied in the MSA, which uses only one necessary addition operation, but at a cost of a more complicated physical structure (Figure 4, Figure 7 and Figure 10).

The general principle of conversion in the FSMSA and the MSA may seem very similar, because both of the variants compensate the overestimation (*R* > *S*) by increasing the source signal *S* (*S_k_* < *S_k_*_+ 1_) rather than decreasing the reference signal *R* (OSA variant). However, from the practical point of view, the FSMSA by definition is more expensive. The additional reference elements entail the consequence of using more hardware resources and consuming more energy. On the other hand, controlling one complex digital-to-analog converter (Figure 10) rather than two simple ones (Figure 7) may be more complicated and bring additional problems. Despite the fact the FSMSA is more expensive than the MSA, there are Time-to-Digital Converters solutions based on the variant [7].

The next difference consists in the relation between the measured input value *X_IN_* and the characteristics of the reference signal *R* and the source signal *S*. In the OSA algorithm, during the conversion process, the measured input value *X_IN_* directly affects the value of the reference signal *R* at each step, while the source signal *S* is constant and equal to the measured input value *X_IN_*. In the FSMSA the reference signal *R* is totally independent of the measured input value *X_IN_*. Its monotonic characteristic is identical in every conversion process. Possible overestimation (*S* < *R*) is compensated by the additional reference elements *A_n_*_−1_, …, *A*_0_, so in this algorithm the source signal *S* is dependent on the measured input value *X_IN_*. In the MSA variant the characteristics of both signals *S* and *R* are related to the measured input value *X_IN_* as the algorithm uses only necessary addition operation, which can be applied to both signals *S* and *R*.

Another parameter, which is differed by the presented models is the last step of the conversion process. For all of the algorithms it is used to evaluate the least significant bit *b*_0_ in the digital output word *b_n_*_−1_, …, *b*_0_. Even though it is a simple comparison of the source signal *S* and the reference signal *R*, it is proceeded within a different operation. The OSA variant compares the signals in order to decide if the reference signal *R* should be reduced or not. The FSMSA, on the contrary, decides whether the source signal *S* should be increased. Thus, both of the algorithms check if any last change of the current state is needed. Finally, the MSA does not allow for any additional correction (reduction of reference signal *R* or increment of the source signal *S*). The last step of this binary SA variant is reduced to the final comparison of the signals, which determines the value of the least significant bit *b*_0_.

Determining the equivalent of the measured input value *X_IN_* is also different, which is clearly visible using the pan balance model. In the OSA it is represented by a subset of the reference elements left on the pan *R* (Equation (2)). There is no necessity of performing any additional mathematical operations. In the FSMSA it can be evaluated as the difference between the total weights of the reference elements accumulated on the pans (Equation (3)). However, at the end of every conversion process the value of the elements accumulated on the pan *R* is always the same and equal to the sum $\overset{n-1}{\underset{k=0}{\sum }}\left(R_{k}\right)$, so the equation can be simplified to (4). Finally, the MSA not only perform a mathematical operation, but also includes the final deflection of the pan balance (Equation (6)), so in this SA algorithm the evaluation equation is the most complicated. Of course, the above-mentioned expressions just make a comment to the presented pan balance model and in practical implementations of the ADCs there is no need for any further mathematical operations at the end of the conversion. The bits in the output digital word *b_n_*_−1_, …, *b*_0_ can be successively evaluated at each step of the conversion process [6,7,10,11].

The application models also differ significantly. From the presented circuits, the OSA model (Figure 4) is the simplest one as the algorithm itself uses relatively simple technique to approximate the measured input value *X_IN_*. In the FSMSA algorithm the model is expanded by the additional digital-to-analog converter DAC_S (Figure 7). Such modification increases the capability of the ADCs using this SA algorithm, but at a cost of more demanding implementation. The additional element DAC_S can increase the occupied area of the ADC. In addition, controlling two digital-to-analog converters separately requires more expanded control logic SAR. Finally, the model of the MSA (Figure 10), similarly to the OSA, uses only one digital-to-analog converter DAC_SR, but this element is much more complex, which obviously also directly affects the implementation.

**Figure 11:**
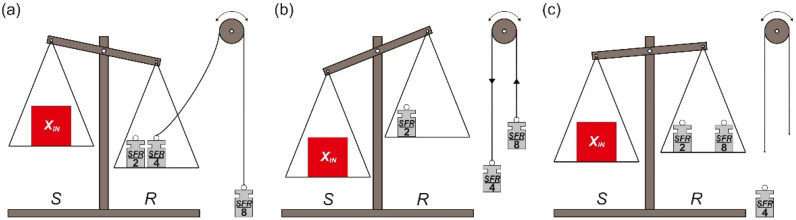
Illustration of the energy-recovery mechanism in the OSA: (**a**) the overestimation; (**b**) energy-recovering compensation; (**c**) compensated overestimation.

The specific character of each SA method defines the energy required to accomplish the conversion process [12]. In reference to the pan balance model, the consumed energy can be presented in such a way that the energy required to place the reference element *R_k_* or *A_k_* (in case of the FSMSA variant) on the pan balance equals *E_k_*. On the above assumption, the total energy *E_T_* required to convert the measured input value *X_IN_* using the OSA algorithm equals:

(7) $$E_{T}=\left(2^{n}-1\right)\cdot E_{0},$$

because all of the reference elements *R_n_*_−1_, …, *R*_0_ have to be placed on the pan *R* regardless of the measured input value *X_IN_*. However, as some of the reference elements are placed and in the next step removed, such elements can be used to lift the subsequent reference element (Figure 11). It means that part of the energy can be recovered, so to some extent the OSA can be implemented as an energy-recoverable ADC. A specific implementation of this idea is presented in [13,14,15]. For relatively small measured input value *X_IN_* (*X_IN_* < *R*_0_) the overestimation (*S* < *R*) occurs at each step of the conversion process. In such case, the total consumed energy is limited only to the placement of the biggest reference element *R_n_*_−1_ on the pan *R*. The energy required to lift each subsequent reference element *R_k_* (*k* = *n* − 2, *n* − 3, …, 0) can be compensated by the previous one *R_k_*_+1_ (Figure 11b). As a result, the total consumed energy *E_OSA_* in the optimized OSA algorithm varies in range:

(8) $$\begin{array}{cc} \left(2^{n-1}\right)\cdot E_{0} & \leq E_{OSA}\leq \left(2^{n}-1\right)\cdot E_{0}\\ \frac{E_{T}+E_{0}}{2} & \leq E_{OSA}\leq E_{T} \end{array}$$

The FSMSA uses two sets of the reference elements and one of them is always fully used (on the reference pan *R*). The expend of the other subset (used on the source pan *S*) depends on the measured input value *X_IN_*. When it is smaller than the reference element *R*_0_ all of the additional reference elements *A_n_*_−1_, …, *A*_0_ are placed on the pan *S* at the end of the conversion process. In contrary, if the measured input value *X_IN_* is relatively high (*X_IN_* ≥ (*SFR* − *R*_0_)), none of the additional reference elements *A_n_*_−1_, …, *A*_0_ is used. In result, the total consumed energy *E_FSMSA_* in the FSMSA algorithm varies in range:

(9) $$\begin{array}{cc} \left(2^{n}-1\right)\cdot E_{0} & \leq E_{FSMSA}\leq 2\cdot \left(2^{n}-1\right)\cdot E_{0}\\ E_{T} & \leq E_{FSMSA}\leq 2\cdot E_{T}. \end{array}$$

The MSA, as the OSA, uses only one set of the reference elements *R_n_*_−1_, …, *R*_0_. However, as in the FSMSA, none of the reference elements *R_n_*_−1_, …, *R*_0_ is removed during conversion process. In result, regardless of the measured input value *X_IN_*, the amount of consumed energy *E_MSA_* in the MSA variant always equals:

(10) $$E_{MSA}=\left(2^{n}-1\right)\cdot E_{0}=E_{T}.$$

The above Equations (8)–(10) show that the maximum of the energy *E_OSA_* consumed by the optimized OSA is equal to the minimum of the energy *E_FSMSA_* consumed by the FSMSA variant. It stems from the fact that the FSMSA uses the additional set of the reference elements *A_n_*_−1_, …, *A*_0_. The MSA consumes always the same amount of energy, which is exactly equal to the maximum of *E_OSA_* and the minimum of *E_FSMSA_*. Furthermore, the value of *E_MSA_* is the only one, which *E_OSA_* and *E_FSMSA_* have in common. It is a consequence of including selected OSA and FSMSA properties in the MSA algorithm.

The above conclusions are summarized in the table below (Table 1):

## 4. Conclusions

In this paper three algorithms of the binary successive approximation method have been distinguished. The following names, relating to their properties, have been proposed: oscillating successive approximation (OSA), full-scale monotonic successive approximation (FSMSA) and monotonic successive approximation (MSA). The distinction was introduced in order to indicate the main differences between the algorithms, which directly affect the final application.

Undoubtedly the most common successive approximation algorithm is the OSA variant. In addition, it is often incorrectly considered to be the only one. The most important difference between the SA algorithms consists in the ability to direct conversion of non-removable physical quantities such as time intervals. Both the FSMSA and the MSA variants are capable of performing such operation, while in case of the OSA it is impossible. Nevertheless, as mentioned above, conversion of the non-removable values applied as the OSA variant is still possible using indirect conversion. Unfortunately, this entails the necessity of extending the whole conversion procedure by prior preconversion process, which is inherently associated with additional measurement error (uncertainty).

Another important issue raised in this paper is the energy demand, which is imposed by the unique character of conversion in each SA variant. It was shown that its general tendency can be successfully estimated using only such simple models.

Presented systematization of the binary SA algorithms, the specific differences between them, and finally the energy consumption allow for correct identification of the applied SA algorithm and its selected properties, which can be essential in some applications.

## Figures and Tables

**Table 1:** Comparison of the binary SA algorithms.

Parameter	Oscillating Successive Approximation (OSA)	Full-Scale Monotonic Successive Approximation (FSMSA)	Monotonic Successive Approximation (MSA)
Ability for direct conversion of non-removable physical quantities	No	Yes	Yes
Number of conversion steps	(*n* + 1)	(*n* + 1)	(*n* + 1)
Varied pattern (character) of the reference signal *R*	Yes	No	Yes
Varied pattern (character) of the source signal *S*	No	Yes	Yes
Operation at the last step	Optional reduction of the reference signal *R*	Optional increase of the source signal *S*	Determining the deflection of the pan balance
Equivalent of the measured input value *X_IN_*	Reference elements on the pan *R* (Equation (2))	Difference between the reference elements on the pan *R* and the additional elements on the pan *S* (Equation (3))	Difference between the reference elements on the pan *R* and the reference elements on the pan *S* (Equation (6))
Complexity of implementation	1 comparator, 1 simple DAC, 1 simple SAR.	1 comparator, 2 simple DACs, 1 complex SAR.	1 comparator 1 complex DAC, 1 complex SAR.
Energy consumption	$\langle \frac{E_{T}+E_{0}}{2},E_{T}\rangle$	$\langle E_{T},2\cdot E_{T}\rangle$	$E_{T}$
Examples of implementation	[16,17,18]	[7]	[19,20,21,22,23]
